# Unpacking Inequities in ADHD Diagnosis: Examining Individual-Level Race/Ethnicity and State-Level Online Information-Seeking Patterns

**DOI:** 10.1007/s10488-023-01259-w

**Published:** 2023-03-17

**Authors:** Xin Zhao, Timothy Hayes, Adela C. Timmons, Wensong Wu, Stacy L. Frazier

**Affiliations:** 1 Department of Medicine, University of California at Irvine, Irvine, USA; 2 Department of Psychology, College of Arts, Sciences, & Education, Florida International University, Miami, USA; 3 Department of Psychology, College of Liberal Arts, University of Texas at Austin, Austin, USA; 4 Department of Mathematics and Statistics, College of Arts, Sciences, & Education, Florida International University, Miami, USA

**Keywords:** State variation, Racial Inequities, ADHD, Google trends, Latinx, Youth of color

## Abstract

Attention-deficit/hyperactivity disorder (ADHD) is a prevalent, persistent, and costly mental health condition. The internet is an increasingly popular source for information related to ADHD. With a nationally representative sample (2018 NSCH), we aimed to separate individual- and state-level effects to examine inequities in ADHD diagnoses. We extracted state-level relative search volumes using “ADHD,” “ADHD treatment,” “ADHD medication,” and “ADHD therapy” from Google Trends, and sociodemographic and clinical variables from the 2018 National Survey of Children’s Health (N = 26,835). We examined state variation in ADHD-related information-seeking and applied multilevel modeling to examine associations among individual-level race/ethnicity, state-level information-seeking patterns, and ADHD diagnoses. Online information seeking related to ADHD varies by state and search term. Individual-level racial/ethnic background and state-level information-seeking patterns were associated with ADHD diagnoses; however, their cross-level interaction was not significant. This study adds to the strong body of evidence documenting geographical variation and diagnostic disparity in mental health and the growing literature on the impact of the digital divide on population health, indicating an urgent need for addressing inequities in mental health care. Increasing public interest in and access to empirically supported online information may increase access to care, especially among people of color.

Attention-deficit/hyperactivity disorder (ADHD) is a persistent, costly, and prevalent neurodevelopmental disorder, more commonly diagnosed in males (Barkley, 2018). ADHD is characterized by cross-setting and often long-term functional impairments (Barkley, 2018; Gordon & Fabiano, 2019; Kuriyan et al., 2013; Pelham et al., 2020), incurring substantial economic burden (Chhibber et al., 2021; Doshi et al., 2012). National surveys report that youth of color were less likely to receive an ADHD diagnosis compared to White youth across the developmental span (Coker et al., 2016; Danielson et al., 2018; Morgan et al., 2013). Yet, a recent meta-analysis reveals that youth from disadvantaged socioeconomic backgrounds are at higher risk for internalizing and externalizing symptoms (Peverill et al., 2021).

Documenting the true prevalence of ADHD remains challenging in light of problems of overdiagnosis (e.g., following quick screening rather than full evaluation incorporating multi-informant and multi-method data given limited resources) and underdiagnosis (e.g., reflecting inequities in healthcare and education systems). State-level diagnostic prevalence of ADHD varies largely (Danielson et al., 2022; Fulton et al., 2015; Visser et al., 2014). Additionally, inequities in diagnoses have been attributed in part to biases from teachers and clinicians, structural racism, imbalanced community resources, insurance status, stigma, psychological literacy, sociocultural expectations, funding, and policy that influence service seeking for families of youth (Alvarez et al., 2021; Fadus et al., 2020; Fulton et al., 2009, 2015; Reardon et al., 2017). Persistent inequities highlight the need for understanding these ethnocultural disparities in care.

Disparities in diagnosis and care begin earlier in the help-seeking sequence, as described by Eiraldi and colleagues (2006) in the ADHD help-seeking behavior model: problem recognition, decision to seek help, service selection, and service utilization (Eiraldi et al., 2006; Zhao et al., 2022). Online health information may be particularly important for the first step of problem recognition and is often the first step toward seeking professional help (Eiraldi et al., 2006). Race/ethnicity, income, insurance, unemployment, and geography have been linked to both information seeking and service utilization for ADHD. Importantly, the internet is increasingly popular as a source for ADHD-specific information among parents (Bussing et al., 2012; Sage et al., 2018), youth (Bussing et al., 2012), and teachers (Akram et al., 2009).

In fact, given the increasing accessibility of the internet in the US, more than 80% of adults search for health information online (Jacobs et al., 2017). Internet searching has significantly increased with access to smartphones, which have become more widely available to historically marginalized families (Statcounter, 2020). Access to and utilization of health information online are partly associated with patients’ and/or caregivers’ emotional status (e.g., elevated health anxiety; Brown et al., 2020), decisions (e.g., deciding to visit a professional; Yu et al., 2019), behaviors (e.g., more frequent physician visits; Brown et al., 2020), and preferences [e.g., for patient-centered care; Baldwin et al., 2008)]. For example, in a hospital waiting room in Canada, 80% of caregivers (n = 143) reported starting with a public search engine (e.g., Google) when seeking health-related information for their child (Pehora et al., 2015), highlighting the importance and opportunity of online information to influence evaluation- and treatment-seeking patterns among families.

Emerging studies in the last decade have incorporated online forum and/or search engine data to examine the patterns, content, and impact of online information-seeking behaviors (Rosenblum & Yom-Tov, 2017; Terbeck & Chesterman, 2012). Prior research on temporal trends of online information seeking related to ADHD suggested that public search interest fluctuated with media and advocacy events using monthly Google Trends data (Zhao et al., 2022). Additionally, state-level Google Trends data point to geographical variation related to online information-seeking patterns (Arora et al., 2019; Mavragani et al., 2018). For instance, Google Trend analyses with keywords “prostate cancer” and “prostate biopsy” revealed that search interest was highest in South Carolina and lowest in Hawaii (Rezaee et al., 2019), highlighting the uneven geographical distribution of search interest and, in turn, possible new avenues for understanding inequity related to seeking and receiving health care. Geographical variation in mental health online information seeking is largely unknown.

## What We Know and Don’t Know and the Present Study

We know that geographical variation and racial/ethnic inequities are consistently documented for ADHD diagnoses (Danielson et al., 2018, 2022; Morgan et al., 2013). We also know that the internet is an increasingly popular source for health information in general (Jacobs et al., 2017), and ADHD-related information specifically (Akram et al., 2009; Bussing et al., 2012; Kubb & Foran, 2020; Sage et al., 2018; Terbeck & Chesterman, 2012). We don’t yet know to what extent *state-level* online information-seeking patterns (i.e., differences in relative search interest in ADHD) vary and relate to *individual-level* ADHD diagnoses among youth. Specifically, the impact of income, insurance/benefits, and employment on access to mental health information seeking and service utilization may vary corresponding to variation in federal and states’ legislative landscape (Hoagwood et al., 2018). Relatedly, state-level variation in racial disparities (e.g., housing stability) (United Health Foundation, 2021) may relate to differences in perceived need for mental health information and diagnosis, particularly among racial/ethnic minority groups. To date, however, the intersectionality between individual racial/ethnic background and state-level information seeking and their impact on diagnostic disparity has received little empirical attention.

Hence, the present study utilized large-scale publicly available sources (i.e., Google Trends, National Survey of Children’s Health [NSCH]) and multilevel analyses to examine geographical variation specific to online information seeking related to ADHD. Specifically, we were interested in the degree to which population-level findings across states were related to individual-level diagnostic and sociodemographic information. We applied multilevel modeling to examine 3 hypotheses: (1) youth of color will be less likely to receive ADHD diagnoses than White youth (reference group), (2) state-level online information seeking (state variation of Google search interest) related to ADHD will be associated with ADHD diagnoses, and (3) there will be a cross-level interaction between individual-level race/ethnicity and state-level online information-seeking patterns, such that youth of color will be even less likely to receive an ADHD diagnosis when residing in a state with less relative search interest in ADHD.

## Methods

Data from public repositories do not require approval from Institutional Review Board or collection of informed consent. We extracted, integrated, and analyzed data in R 4.0.3 (R Core Team, 2020).

### Data Source and Sample

#### Google Trends

Our measures of information seeking included state-level relative search volumes (RSVs) for 50 states and Washington DC (hereafter, N = 51) for “ADHD,” “ADHD treatment,” “ADHD medication,” and “ADHD therapy” in 2018 from Google Trends (https://trends.google.com/trends/?geo=US). State-level RSVs were standardized and scaled, after filtering duplicate searches (repeated searches from the same person over a short time). They represent the scaled proportion of searches containing a specified (set of) search term(s); in this study, state-level RSVs represent the number of searches for “ADHD” divided by the number of all searches *within each state* and scaled in relevance to all other states. RSVs account for differences in internet access and population size, ranging from 0 (very low search interest within the state) to 100 (representing the state with the highest search interest out of all 50 states and Washington DC); the state with the highest search interest out of all states has an RSV = 100.

#### National Survey of Children’s Health (NSCH)

We extracted data from the 2018 National Survey of Children’s Health (NSCH; Child and Adolescent Health Measurement Initiative [CAHMI], 2019). The study did not require Institutional Review Board approval or collection of informed consent, as data are publicly available. The corresponding author completed the data use agreement from the Data Resource Center and CAHMI. A national representative sample of caregivers of youth aged 0 to 17 years completed questionnaires via mailed packets and online surveys to provide demographic and health information; one child was randomly selected in families with multiple children. Details of the survey instruments are available at https://www.childhealthdata.org/learn-about-the-nsch/survey-instruments. In the current study, 26,205 caregivers of youth aged 3 to 17 years old provided a valid response regarding the status of their child’s ADHD diagnosis. Unweighted demographic characteristics (i.e., race/ethnicity, poverty status, highest education in household, child sex, child age) by these three conditions in the current sample are presented in Table 1. Note the federal poverty level variable had 16.03% missing values in 2018 in the original data collection and the Census Bureau imputed the missing values in its publicly available dataset. We utilized the variable generated using a single imputation value from the 2018 data file (“FPL_I1”) based on US Census guidelines. Additional details regarding the impact of missing values on population count estimates are available on the source website (https://www.childhealthdata.org/learn-about-the-nsch/). The number of respondents averaged 513 per US state and Washington DC, ranging from 396 in DC to 672 in Arkansas. Individual responses (level 1) were nested in states of residency (50 states and Washington DC; level 2).

**Table 1:** Sociodemographic Information by ADHD diagnostic condition

	Does not have ADHD (n = 23,295)		Ever told, but does not currently have ADHD (n = 233)		Currently has ADHD (n = 2,677)		*X*^*2*^ *(df)*	*p*
	*M* or *n*	*SD* or *%*	*M* or *n*	*SD* or *%*	*M* or *n*	*SD* or *%*		
Race/Ethnicity							105.32 (8)	< 0.001
Latinx, Hispanic	2815	12%	25	11%	248	9%		
White, non-Hispanic	15,998	67%	165	71%	1981	74%		
Black, non-Hispanic	1491	6%	19	8%	193	7%		
Asian, non-Hispanic	1209	5%	5	2%	38	1%		
Other/Multi-racial, non-Hispanic	1782	7%	19	8%	217	8%		
Poverty Status							59.61 (6)	< 0.001
0–99% FPL	2641	11%	30	13%	415	16%		
100–199% FPL	3749	16%	48	21%	483	18%		
200–399% FPL	7177	30%	72	31%	783	29%		
400% FPL or more	9728	41%	83	36%	996	37%		
Highest education in the household: reference level							96.97 (6)	< 0.001
Less than high school	633	3%	7	3%	67	3%		
High school or GED	3097	13%	41	18%	448	17%		
Some college or technical school	5485	23%	67	29%	788	29%		
College and above	14,080	59%	118	51%	1374	51%		
Child sex: Male	11,699	49%	159	68%	1845	69%	360.51 (2)	< 0.001
Child age	10.48	4.48	14.07	2.97	12.24	3.38		

The federal poverty level variable was generated using a single imputation value from the 2018 data file (FPL_I1) based on US Census guidelines. Details regarding the impact of missing values on population count estimates is available on the source website (https://www.childhealthdata.org/learn-about-the-nsch/)

*ADHD* Attention-deficit/hyperactivity disorder, *FPL* federal poverty level, *GED* General Educational Development, *M* Mean, *SD* standard deviation. *df* degree of freedom

### Analytical Plan

Applying hierarchical multilevel modeling (MLM) regression models using the “glmer” function in the “lme4” package in R (Bates et al., 2015), we assessed the fit of each step after controlling for initial (earlier) steps of variables. MLM allows for modeling between-state variation and partitioning level-1 (individual-level) and level-2 (state-level) effects. All categorical variables were dummy coded; White was the reference group for child race/ethnicity. All level-1 predictors (including continuous and dummy coded categorical variables) were cluster-mean centered (Enders & Tofighi, 2007). We present level-specific descriptive statistics in Appendix 1. The order of entry of sets of independent variables into the regression model was predetermined to test for hypotheses.

#### Model Specifications

Our first model was a random intercept model with current ADHD diagnosis as the binary criterion variable and all level-1 predictor variables, including the main variable of interest (child race/ethnicity) and control variables (i.e., household income, highest education in the household, child sex, child age). Our second model included all variables in Model 1 and online search interest (Google RSV) as the level-2 predictor. Our third model added cross-level interactions between race/ethnicity and state-level search interest to the second model. For model comparison, we computed Akaike information criterion (AIC; Akaike, 1974) and Bayesian information criterion (BIC) and conducted *X*^2^ tests. After identifying the best-fitting model, we have conducted additional analyses by adding weights in Model 2 using three weight options: (1) an unscaled weighted model: using the “FWC” variable from the dataset; (2) a scaled weighted model using weights adjusted by a factor that represents the proportion of cluster size divided by the sum of sampling weights within each cluster; (3) a scaled weighted model using weights adjusted by the sum of sample weights within each cluster divided by the sum of squared sample weights within each cluster. Weights were scaled using the “scale_weights” function in the sjstats (version 0.18.0) package in R (Lüdecke, 2020).

### Variables for Multilevel Logistic Regression

#### Level-1 Predictors

Race/ethnicity was the main variable of interest. We controlled for income, highest education in the household, child’s sex, and child’s age in years.

#### Level-2 Predictor

State-level RSVs for 50 states and Washington DC (hereafter, N = 51) are commonly used in the medical field as a metric to reflect geographical variation in public interest and information-seeking behaviors online (Arora et al., 2019; Mavragani et al., 2018). We selected “ADHD” as our search term because (1) results of searching the term “ADHD” include terms like “ADHD diagnosis,” “ADHD treatment,” “ADHD medication,” “ADHD therapy” and (2) the abbreviation of the disorder seems to be more commonly known and used by the public.

#### Dependent Variable

The dependent variable was *current* ADHD diagnosis (1 = Yes, 0 = No). Caregivers were asked, “Has a doctor or other health care provider EVER told you that this child has Attention Deficit Disorder or Attention Deficit/Hyperactivity Disorder, that is, ADD or ADHD?” Caregivers who responded yes to this initial question were asked, “Does this child CURRENTLY have the condition?” Given all level-1 and level-2 predictors were extracted for 2018, we focused on *current* diagnosis in 2018; children without current ADHD diagnoses included those who had never been diagnosed with ADHD (n = 23,295) and those who were ever diagnosed but do not currently have a diagnosis of ADHD (n = 233).

## Results

### Data Visualization: State Variation in Online Information About ADHD

**Fig. 1:**
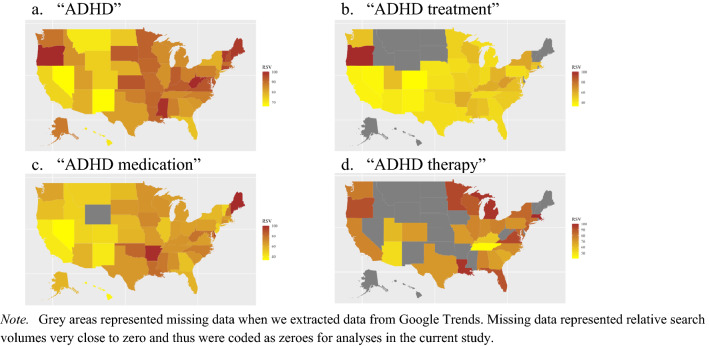
State variations in google trends relative search volumes

State variation in searches for “ADHD” is illustrated in Fig. 1. State-level search interest in “ADHD” was highest in West Virginia and Oregon and lowest in Nevada and New Mexico. Search interest in “ADHD treatment” was highest in Oregon, followed by West Virginia and lowest in Colorado, Nevada, California, and New Mexico. A total of 13 states displayed missing RSVs (grey areas) for “ADHD treatment.” Search interest in “ADHD medication” was highest in Maine and Arkansas and lowest in Nevada and Hawaii. Wyoming displayed missing RSV for “ADHD medication.” Search interest in “ADHD therapy” was highest in Massachusetts, Michigan, and Louisiana, and lowest in Tennessee and Arizona. A total of 22 states displayed missing RSVs for “ADHD therapy.” We coded missing values as zeroes, as they indicated very low search interest.

### Multilevel Modeling

#### Intraclass Correlation and Model Selection

We computed intraclass correlation coefficients^1^ using maximum likelihood estimation. The intraclass correlation (ICC) was 0.01, indicating that residency of state accounted for 1% of the variance in ADHD diagnosis. Research has shown that we can benefit from analyses in the MLM framework when ICC $\ge$ 0.01 (Bliese, 1998). Model comparison is presented in Table 2. Model 2 (*AIC*=16,322, *BIC*=16,543) fit data significantly better than Model 1 (*AIC*=16,336, *BIC*=16,548), *X*^2^ = 15.58, *df* = 1, *p*<.001). Model 3, which added the cross-level interaction to Model 2, did not outperform Model 2, *AIC*=16,331, *BIC*=16,617, *X*^2^ = 7.22, *df* = 8, *p*=.513. Thus, Model 2 (including all level-1 predictors and state-level search interest) was selected as the final model. Parameters of all three models are presented in Table 3. Weighted analyses for Model 2 were presented in Appendix 2. Unscaled weighted analyses (Model 2O) were most different compared to other models. Scaled weighted findings (Model 2 A and Model 2B in Appendix 2) were comparable to unweighted analyses (what is presented in text and Table 2); small inferential differences were found for some covariate variables.

**Table 2:** Results of multilevel modeling predicting current ADHD diagnoses

	Model 1: random intercept with level-1 predictors					Model 2: Model 1 + RSV as a level-2 predictor					Model 3: Model 2 + cross-level interaction				
	Fixed effects					Fixed effects					Fixed effects				
	*Coef*	*OR*	*SE*	*z*	*p*	*Coef*	*OR*	*SE*	*z*	*p*	*Coef*	*OR*	*SE*	*z*	*p*
Intercept	− 2.85	0.06	1.34	− 2.13	0.034	− 2.59	0.08	1.29	− 2.00	0.045	− 3.46	0.03	1.43	− 2.42	0.016

	Within states					Within states					Within states				
Race: reference group = White, non-Hispanic															
Latinx, Hispanic	− 0.39	0.68	0.08	− 5.08	< 0.001	− 0.39	0.68	0.08	− 5.08	< 0.001	− 1.16	0.32	0.70	− 1.64	0.101
Black, non-Hispanic	− 0.24	0.78	0.09	− 2.80	0.005	− 0.24	0.78	0.09	− 2.78	0.005	0.09	1.09	1.12	0.08	0.937
Asian, non-Hispanic	− 1.30	0.27	0.17	− 7.65	< 0.001	− 1.30	0.27	0.17	− 7.64	< 0.001	0.53	1.69	1.53	0.34	0.731
Other/Multi-racial, non− Hispanic	0.00	1.00	0.08	− 0.01	0.996	0.00	1.00	0.08	0.01	0.992	− 0.40	0.67	0.79	− 0.51	0.609
Income: reference = 400% FPL or more															
0–99% FPL	0.40	1.49	0.07	5.54	< 0.001	0.40	1.49	0.07	5.54	< 0.001	0.40	1.49	0.07	5.51	0.000
100–199% FPL	0.17	1.19	0.07	2.62	0.009	0.17	1.19	0.07	2.63	0.008	0.17	1.19	0.07	2.61	0.009
200–399% FPL	0.03	1.03	0.05	0.64	0.520	0.04	1.04	0.05	0.66	0.508	0.03	1.04	0.05	0.65	0.513
Highest education in the household: reference level = college and above															
Less than high school	− 0.05	0.95	0.14	− 0.38	0.705	− 0.05	0.95	0.14	− 0.37	0.708	− 0.05	0.95	0.14	− 0.36	0.722
High school or GED	0.23	1.26	0.06	3.55	< 0.001	0.23	1.26	0.06	3.54	< 0.001	0.23	1.26	0.06	3.54	< 0.001
Some college or technical school	0.30	1.35	0.05	5.83	< 0.001	0.30	1.35	0.05	5.81	< 0.001	0.30	1.35	0.05	5.84	< 0.001
Child sex: reference group = Male	− 0.78	0.46	0.04	− 17.67	< 0.001	− 0.78	0.46	0.04	− 17.68	< 0.001	− 0.78	0.46	0.04	− 17.68	< 0.001
Child age	0.10	1.10	0.01	18.90	< 0.001	0.10	1.10	0.01	18.90	< 0.001	0.10	1.10	0.01	18.89	< 0.001
Latinx, Hispanic*RSV	–	−	−	−	−	−	−	−	−	−	0.01	1.01	0.01	1.10	0.271
Black, non− Hispanic*RSV	–	–	–	–	–	–	–	–	–	–	0.00	1.00	0.01	− 0.30	0.766
Asian, non-Hispanic*RSV	–	–	–	–	–	–	–	–	–	–	− 0.02	0.98	0.02	− 1.20	0.230
Other/Multi-racial, non-Hispanic*RSV	–	–	–	–	–	–	–	–	–	–	0.00	1.00	0.01	0.52	0.604

	Between states					Between states					Between states				
Race: reference group = White, non-Hispanic															
Latinx, Hispanic	− 1.19	0.31	0.42	− 2.80	0.005	− 0.73	0.48	0.43	− 1.72	0.086	− 2.93	0.05	2.69	− 1.09	0.277
Black, non-Hispanic	1.06	2.88	0.55	1.93	0.054	0.81	2.24	0.52	1.54	0.124	4.20	66.56	4.27	0.98	0.326
Asian, non-Hispanic	− 1.09	0.34	0.72	− 1.51	0.130	− 0.65	0.52	0.70	− 0.92	0.356	− 13.85	0.00	8.77	− 1.58	0.114
Other/Multi-racial, non-Hispanic	− 0.19	0.83	0.64	− 0.29	0.771	-− 0.02	0.98	0.62	− 0.04	0.970	11.89	146376.58	8.29	1.43	0.151
Income: reference = 400% FPL or more															
0–99% FPL	− 0.34	0.71	1.32	− 0.26	0.796	0.72	2.06	1.30	0.56	0.578	1.63	5.10	1.46	1.12	0.265
100–199% FPL	− 1.57	0.21	0.95	− 1.65	0.099	− 1.44	0.24	0.91	− 1.57	0.116	− 1.23	0.29	0.94	− 1.30	0.192
200–399% FPL	− 0.78	0.46	0.75	− 1.03	0.301	− 0.05	0.95	0.75	− 0.07	0.945	0.43	1.54	0.88	0.50	0.621
Highest education in the household: reference level = college and above															
Less than high school	6.24	511.79	2.70	2.31	0.021	6.95	1040.70	2.63	2.64	0.008	5.80	330.44	2.78	2.09	0.037
High school or GED	1.70	5.49	1.03	1.66	0.097	0.68	1.98	1.01	0.67	0.501	0.73	2.07	1.03	0.71	0.480
Some college or technical school	0.98	2.66	1.07	0.92	0.359	0.81	2.24	1.04	0.77	0.440	0.48	1.62	1.14	0.42	0.671
Child Sex: Reference group = Male	− 1.21	0.30	1.22	-0.99	0.322	-2.22	0.11	1.20	− 1.86	0.063	− 1.65	0.19	1.46	− 1.13	0.259
Child age	0.10	1.11	0.11	0.94	0.345	-0.02	0.98	0.11	-0.15	0.879	0.00	1.00	0.11	− 0.04	0.968
Latinx, Hispanic*RSV	–	–	–	–	–	–	–	–	–	–	0.03	1.03	0.04	0.81	0.416
Black, non-Hispanic*RSV	–	–	–	–	–	–	–	–	–	–	− 0.04	0.96	0.05	− 0.85	0.396
Asian, non-Hispanic*RSV	–	–	–	–	–	–	–	–	–	–	0.16	1.18	0.11	1.50	0.134
Other/Multi-racial, non-Hispanic*RSV	–	–	–	–	–	–	–	–	–	–	− 0.14	0.87	0.10	− 1.43	0.152
RSV	–	–	–	–	–	0.01	1.01	0.00	3.97	< 0.001	0.02	1.02	0.01	2.02	0.044

	Random effect (variance)					Random effect (variance)					Random effect (variance)				
State (Intercept)	0.002					< 0.001					< 0.001				

*FPL* federal poverty level, *GED* General Educational Development, *RSV* Google Trends state-level relative search volume, *Coef* = coefficient, *OR* odds ratio, *SE* standard error

**Table 3:** Model comparison

	AIC	BIC	logLik	Deviance	X^2^	df	p
Model 1	16,336	16,548	− 8141.8	16,284	–	–	–
Model 2	16,322	16,543	− 8134	16,268	15.575	1	< 0.001
Model 3	16,331	16,617	− 8130.4	16,261	7.2228	8	0.5128

*AIC*  Akaike information criterion, *BIC* Bayesian information criterion, *df* degree of freedom

#### Covariates: Income, Education in the Household, Child’s Sex and Child’s Age

At level 1, youth living in high poverty (0–99% federal poverty level [FPL]: *b* = 0.40, *OR* = 1.49, *SE* = 0.07, *z* = 5.54, *p* < .001; 100–199% FPL: *b* = 0.17, *OR* = 1.19, *SE* = 0.07, *z* = 2.63, *p* = .008) were more likely to have an ADHD diagnosis; such results were not significant in scaled weighted analyses (Appendix 2). Older youth also were more likely to have a current ADHD diagnosis, *b* = 0.01, *OR* = 1.1, *SE* = 0.01, *z* = 18.90, *p* < .001. Compared to male youth, female youth were less likely to have a current ADHD diagnosis, *b*=-0.78, *OR* = 0.46, *SE* = 0.04, *z*=-17.68, *p* < .001. Highest education in household below college (high school or GED: *b* = 0.23, *OR* = 1.26, *SE* = 0.06, *z* = 3.54, *p* < .001; some college or technical school: *b* = 0.30, *OR* = 1.35, *SE* = 0.05, *z* = 5.81, *p* < .001) was, mostly, associated with higher likelihood of ADHD diagnosis for children; however, this association was not reported for families with “Less than high school” education at level 1. Instead, youth were more likely to receive an ADHD diagnosis in states where there was a higher percentage of households with less than high school education (level-2 specific effect), *b* = 6.95, *SE* = 2.63, *z* = 2.64, *p* = .008; and this association was not significant in scaled weighted analyses (Appendix 2).

#### Hypothesis 1: Youth of Color Were Less Likely to Receive ADHD Diagnoses

As shown in Table 2, in Model 2 (best-fitting model), within states (at level 1), Black (*b*=-0.24, *OR* = 0.78, *SE* = 0.09, *z*=-2.80, *p* = .005), Latinx/Hispanic (*b*=-0.39, *OR* = 0.68, *SE* = 0.08, *z*=-5.08, *p <* .001), and Asian (*b*=-1.30, *OR* = 0.27, *SE* = 0.17, *z*=-7.65, *p <* .001) youth were less likely to have a current ADHD diagnosis, compared to White youth, after controlling for poverty status, highest education in household, child’s sex, and child’s age. Compared to White youth (most likely to receive an ADHD diagnosis), Black youth were 22% less likely, Latinx youth were 32% less likely, and Asian youth were 73% less likely to receive an ADHD diagnosis.

#### Hypothesis 2: State-level Online Information Seeking Related to ADHD Predicted Diagnoses

**Fig. 2:**
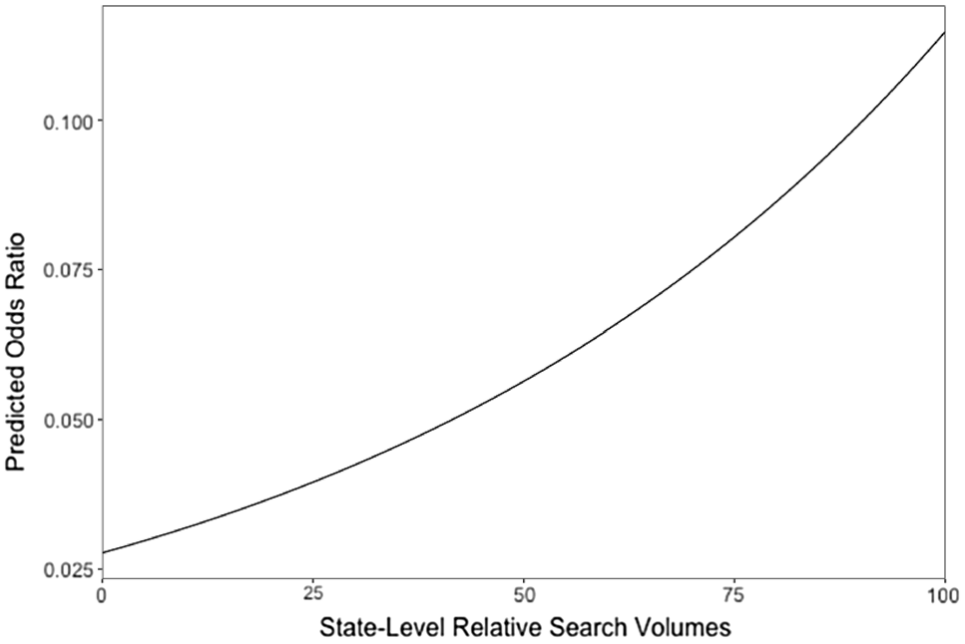
The relationship between state-level relative search volumes and predicted odds ratio

Model 2 (best-fitting) reveals that higher levels of state-level RSV were associated with a higher likelihood of ADHD diagnoses, *b* = 0.01, *OR* = 1.01, *SE* = 0.00, *z* = 3.97, *p* < .001 (Table 2). We computed Rights and Sterba’s suite of multilevel R^2^ (Rights & Sterba, 2019, 2020) and found that this fixed effect (between-level) level-2 variable predicted 1% of the total variance, which represents nearly all of the between-state variance. We also plotted the relationship between state-level RSV (x-axis) and predicted odds ratios holding all other predictors constant at their means (Fig. 2).

#### Hypothesis 3: No Cross-Level Interaction Between Individual-Level Race/Ethnicity and State-Level Online Information-Seeking Patterns

Contrary to our predictions, there was no significant interaction between individual-level (level-1) race/ethnicity and state-level (level-2) search interest in Model 3 (Table 2). Youth were not less likely to receive an ADHD diagnosis when residing in a state with less relative search interest in ADHD.

## Discussion

This study adds to the strong body of evidence documenting racial/ethnic inequities in mental health care and the growing literature on the impact of the digital divide on population health. With a large (N = 26,835) and nationally representative sample (2018 NSCH), we separated individual- and state-level effects to examine inequities in ADHD diagnoses. Specifically, we applied multilevel modeling to examine the extent to which *individual-level* racial/ethnic backgrounds and *state-level* information-seeking variations relate to ADHD diagnosis, after controlling for poverty status, highest education in household, child’s sex, and child’s age. Our hypotheses were partially supported. Results support a large body of data pointing to sociodemographic disparity in ADHD diagnoses. The absence of an interaction between the two suggests the need for individual- and state-level analyses.

Findings may be traced to community outreach efforts in areas with major healthcare systems, such as the Oregon Health and Science University Center for ADHD (opened in 2019) that sparked media coverage and, perhaps, searches for ADHD. Except for Oregon, lower interest in ADHD-related information reflected lower prevalence of ADHD diagnoses in the West. States with more youth with ADHD diagnoses (e.g., West Virginia: 16%) displayed relatively high search interest in ADHD and ADHD treatment. Specific to medication, search interest was high in Maine and low in Nevada, corresponding to their difference in state averages of methylphenidate consumption in 2016 (Piper et al., 2018). Specific to therapy, missing data might reflect very low search interest in most sparsely populated states. Access to clinical research and care may not always motivate online information-seeking behaviors. For example, Hawaii, a state with large-scale initiatives to improve youth mental health system of care (Nakamura et al., 2014), did not display high search interest in any ADHD search term. Possibly, these initiatives were not only for ADHD, and thus did not trigger more interest in ADHD compared to other topics (recall RSVs reflect relative interest rather than search volumes). Specific to medication, results were consistent with lower stimulants per capita reported in Hawaii (Piper et al., 2018). More importantly, we hypothesize parabola-shaped relations between information need and access. Accordingly, access to information may increase to an optimal point that increases the desire for information and thereby motivates searching; however, increased access to information may lead to saturation (e.g., “I know enough”) or confusion (e.g., “My head is swimming”) that tempers searching, thereby forming an *inverted-U curve*.

### Youth of Color Were Less Likely to Have a Current ADHD Diagnosis

Findings revealed that youth of color were less likely to receive an ADHD diagnosis, supporting Hypothesis 1. This result is consistent with national surveys reporting racial/ethnic differences in ADHD prevalence (Coker et al., 2016; Danielson et al., 2018; Morgan et al., 2013) and prior work that underscores sociodemographic and systemic (e.g., racism, oppression) barriers to help-seeking pathways for minority families (Cauce et al., 2002; Eiraldi et al., 2006; Fadus et al., 2020). Given our focus on ADHD diagnosis rather than service utilization in general, findings may apply in particular to the initial problem recognition stage (Gerdes et al., 2014; Haack et al., 2018), which is often the first step toward seeking professional help (Eiraldi et al., 2006). Note Danielson et al. (2018) reported Black youth were more likely to receive an ADHD diagnosis (ever and current), compared to White youth, in the 2016 NSCH (reverse of what we found here in the 2018 NSCH). Perhaps this inconsistency resulted from differences in analytical procedures (e.g., model specification and covariates). Alternatively, this finding may be considered in the context of policy changes corresponding to years of data collection; notably, efforts to dismantle the Affordable Care Act that began in 2016 reversed improvements in insurance coverage for youth and mitigation in racial/ethnic disparities (Ortega et al., 2020).

Findings on covariates are consistent with prior literature. Males and older youth were more likely to have an ADHD diagnosis, consistent with results from the 2016 NSCH (Danielson et al., 2018). Youth living in high poverty (family income < 200% FPL) and in households with lower educational attainment (i.e., parents with high school diploma or GED; some college or technical school) were more likely to receive an ADHD diagnosis. Such results can be interpreted in the context of four lines of literature: (1) implicit biases, structural racism, and inequities in school discipline and community resources led to disparities in ADHD diagnoses (Fadus et al., 2020); (2) youth from disadvantaged socioeconomic backgrounds may be at higher risk for behavioral problems and socioemotional impairments (Peverill et al., 2021); (3) caregivers of youth with ADHD experience loss of work productivity and income (Zhao et al., 2019); and (4) ADHD is chronic and heritable, associated with long-term educational and occupational impairments (Gordon & Fabiano, 2019; Kuriyan et al., 2013). Notably, the small inferential differences in scaled weighted analyses and unweighted analyses may be associated with cluster sizes. Residing in a household with less than high school education did not relate to a child’s ADHD diagnosis at the individual level (recall level-1 analyses); this also may reflect small and uneven sample sizes across states under this category.

### State-Level Online Information Seeking Predicted Individual Diagnosis

State-level search interest in ADHD positively predicted ADHD diagnoses, after controlling for level-1 predictors (i.e., race/ethnicity, poverty status, highest education in household, child’s sex, and child’s age), supporting hypothesis 2. Note the relatively low OR = 1.01 (i.e., the multiplicative change resulting from a unit increase in the predictor, holding all other values constant) may result from (1) the small sample size at level 2 (n = 50 US and Washington DC) and (2) relatively low variability of data at level 2 (ICC = 0.01). Despite the small changes of OR, online search interest explained almost all of the variance at level 2. Also, the predicted odds ratios corresponding to RSV (range = 0 to 100) ranged 0.025 to 0.125 (odds ratios), holding all other model predictors constant at their means (Fig. 2). Thus, these findings highlight the potentially important association between information-seeking online and ADHD diagnosis, above and beyond sociodemographic inequities, suggesting the need for additional evaluation. Although Google searching for “ADHD” is not exclusively applicable to youth-serving settings, we found a significant association between state-level search interest and parent-reported current ADHD diagnosis for their child in the 2018 NSCH. This result is consistent with prior studies reporting that the internet has become an increasingly popular source for ADHD-related information among youth, parents and teachers (Akram et al., 2009; Bussing et al., 2012; Sage et al., 2018) and most service-seeking parents start with public search engines (Pehora et al., 2015).

### No Interaction Between Race/Ethnicity and Information Seeking

There was no interaction between individual-level racial/ethnic background and state-level information-seeking patterns, contrary to hypothesis 3, which may be due to the lack of variance at level 2 (ICC = 0.01). Specifically, the state-level online information-seeking variation did not affect the odds that youth of color would have a current ADHD diagnosis over and above other included characteristics. The variable (Google Trends RSVs) measuring information-seeking patterns is not available at the individual level (Mavragani et al., 2018); recall state-level RSVs are scaled values displaying *relative* interest in “ADHD” compared to all other states rather than the *absolute* search volumes of identifiable individuals. Thus, we were unable to examine temporal relations between information-seeking behaviors and sociocultural beliefs at the individual level, which may affect seeking and/or reporting an ADHD diagnosis.

### Limitations and Future Research

First, our analyses were conducted for 2018 at the state level, and thus may not generalize to diagnosis and information seeking during (or after) the Covid-19 global pandemic. States and school districts responded differently to the Covid-19 outbreak, regarding structure and support for virtual learning, and timeline of mandating school closures and reopening; thus, geographic variation in access to mental health information and academic support may have increased (Storey & Slavin, 2020). Exacerbated symptoms and functional impairment experienced by youth with ADHD have been reported in early-pandemic survey studies (Becker et al., 2020; Breaux et al., 2021; Sibley et al., 2021), indicating high information need. Additionally, diminished teacher-parent communication and school-based support during remote learning (Breaux et al., 2021) suggested limited opportunities for gathering information from teachers and schools (and thus a potentially higher need for related information online). Given these changes in information need and access, the internet may have become even more popular and important for ADHD-related information for families. Hence, there will be a substantial need for understanding the relations between online information-seeking behaviors and mental health care since 2020, especially among underserved populations (e.g., those with limited digital access and psychological literacy). Thus, we hypothesize that future studies may find a stronger association between online information-seeking and ADHD diagnosis.

Second, despite the significant associations reported at individual and state levels, we note the nature of *association* rather than *causality* in the current study. Data are cross-sectional; thus, we cannot draw conclusions for specific individual processes and pathways, which is a common limitation for Google Trends data (Mavragani et al., 2018). Possibly, a multilevel mediation may be detected should longitudinal data (e.g., search behaviors of individual families over time) become available. A close examination of parameter changes from Model 1 and Model 2 provided preliminary evidence for this hypothesis. Model 1 also indicated that residing in states with more Hispanic families is associated with a lower likelihood of having an ADHD diagnosis. This association was not significant in Model 2 (when state-level search interest was added as a level-2 predictor), suggesting potential mediation rather than moderation (which we tested in the current study). Additionally, the absence of an interaction between *individual-level* racial/ethnic backgrounds and *state-level* information-seeking variations suggests the need for level-specific analyses, such as individual processes and state policies; for instance, future research may examine information-seeking and diagnosis-seeking processes at the individual level (e.g., collecting both search and decision-making data from individual families) and their association with specific state-level policies (i.e., Medicaid).

Third, we did not include information about cultural identities beyond race and ethnicity – such as nation of origin, immigration status, enculturation and language preference – due to concerns of collinearity. However, it is important to consider intersectionality (Hays, 2016), and especially to avoid monolithic conclusions about how simply identifying with a racial group may relate to health information seeking and decision making. For example, not every family knows and uses the word “ADHD” during Google searches; Spanish-speaking families may search “TADH (Trastorno por Déficit de la Atención con Hiperactividad).” Thus, there may be a language-specific effect on information-seeking behaviors. Future research could benefit from exploring more culture-relevant variables as they relate to online information-seeking behaviors, ideally in ethnoculturally and linguistically diverse samples.

Fourth, we are not able to draw conclusions specific to seeking *empirically supported* information and care. Increasingly, studies have demonstrated that quality of health information online varies considerably (King et al., 2021); thus, a layperson audience may experience challenges in evaluating the quality of online information, receiving and/or utilizing empirically unsupported information (Swire-Thompson & Lazer, 2020). Our variable for information-seeking patterns (state-level RSVs from Google Trends) was extracted for the search term “ADHD,” which captured active information seeking online, possibly reflecting accessing *popular* (decided by Google’s algorithms), but not necessarily high-quality, information on the internet. Additionally, our analyses focused on current ADHD diagnoses (dependent variable); the NSCH also includes information about ADHD medication use and receipt of psychosocial treatment for ADHD, which can be analyzed to examine the multilevel relations among individual demographic characteristics, state-level information seeking, and service utilization.

Fifth, the NSCH did not ask caregivers to report the circumstances of their child’s ADHD diagnosis, such as who gave the diagnosis (e.g., psychologist, psychiatrist, pediatrician) or on what information it was based (e.g., multi-informant and multi-method data, following science-informed practice, or brief screening). Gold-standard research assessment recommendations for ADHD (which require multi-informant data; Pelham, Jr. et al., 2005) are often displaced by quick screening in routine care (NSCH assesses routine care) because most pediatric visits last < 25 min (https://www.statista.com/statistics/697310/pediatricians-minutes-with-patients-us/). More research is needed to understand whether and how online information seeking helps or hinders *science-informed* decision-making processes, particularly for marginalized populations.

Sixth, we do have information about the design variables and sample weights of Google Trends state-level RSVs (level 2 predictor). Carle (2009) utilized simulations to demonstrate “a need for *some type* of scaling if using weights, especially with small cluster sizes. If one cannot scale the weights and include them properly in the estimation, analyzing the data *without* weights provides the next best option.” Our unweighted analyses were comparable to scaled weighted analyses incorporating level-1 weights; replication analyses are needed should design variables and sample weights for Google Trends data become available in the future.

## Conclusion

Persistent racial/ethnic inequities warrant systematic changes in policy and clinical care that can attend to the needs of underserved communities. The digital divide adds complexity to persistent racial/ethnic and socioeconomic inequities in ADHD diagnosis; we found information-seeking patterns varied across states and by search terms. Equitable online information may increase access to mental health diagnoses and in turn, resources and services. Future research is needed for understanding individual pathways and the extent to which online information inspires seeking *science-informed* care. There is potential to leverage public search engine data to enhance access to empirically supported mental health information and care.

## Data Availability

All data are publicly available.

## Appendix 1

**Table:** Descriptive statistics by level

	*M*	*SD*
Level 1 (individual-level, within-cluster)		
Race: Reference group = White, non-Hispanic		
Latinx, Hispanic	0	0.31
Black, non-Hispanic	0	0.24
Asian, non-Hispanic	0	0.21
Other/Multi-racial, non-Hispanic	0	0.26
Income: Reference = 400% FPL or more		
0-99% FPL	0	0.32
100-199% FPL	0	0.37
200-399% FPL	0	0.46
Highest education in the household: reference level = college and above		
Less than high school	0	0.16
High school or GED	0	0.34
Some college or technical school	0	0.43
Child sex: Reference group = Male	0	0.02
Child age	0	0.50
Level 2 (state-level/between-cluster)		
Race: reference group = White, non-Hispanic		
Latinx, non-Hispanic	0.12	0.10
Black, non-Hispanic	0.06	0.07
Asian, non-Hispanic	0.05	0.05
Other/Multi-racial, non-Hispanic	0.08	0.05
Income: reference = 400% FPL or more		
0-99% FPL	0.12	0.04
100-199% FPL	0.16	0.03
200-399% FPL	0.31	0.06
Highest education in the household: Reference level = college and above		
Less than high school	0.03	0.01
High school or GED	0.14	0.04
Some college or technical school	0.24	0.04
Child sex: Reference group = Male	0.48	0.02
Child age	0.22	0.02
RSV	86.27	8.65

Level-1 means = means of cluster-mean-centered scores. Level-2 means = cluster means. Clusters = states. FPL = federal poverty level. RSV =Google Trends state-level relative search volumes

## Appendix 2

**Table:** Weighted analyses for model 2

	Model 2O (Original weights)					Model 2A (Scaled weight A)					Model 2B (Scaled weights B)				
	Fixed effect					Fixed effect					Fixed effect				
	*Coef*	*OR*	*SE*	*z*	*p*	*Coef*	*OR*	*SE*	*z*	*p*	*Coef*	*OR*	*SE*	*z*	*p*
Intercept	− 1.92	0.15	1.53	− 1.26	0.209	− 2.49	0.083	1.59	− 1.57	.117	− 2.56	0.08	1.87	− 1.37	.170

	Within states					Within states					Within states				
Race: reference group = White, non− Hispanic															
Latinx, Hispanic	− 0.53	0.59	0.00	− 381.71	< .001	− 0.59	0.56	0.08	− 7.68	< .001	− 0.59	0.55	0.10	− 5.78	< .001
Black, non-Hispanic	− 0.15	0.86	0.00	− 102.93	< .001	− 0.19	0.83	0.07	− 2.59	0.01	− 0.19	0.83	0.10	− 1.95	.052
Asian, non-Hispanic	− 1.61	0.20	0.00	− 374.42	< .001	− 1.68	0.19	0.25	− 6.75	< .001	− 1.67	0.19	0.33	− 5.03	< .001
Other/Multi-racial, non-Hispanic	− 0.01	0.99	0.00	− 3.88	< .001	0.006	1.01	0.09	0.07	.942	0.02	1.02	0.12	0.13	.895
Income: Reference = 400% FPL or more															
0-99% FPL	0.18	1.20	0.00	114.69	< .001	0.416	1.52	0.07	5.58	<.001	0.42	1.53	0.10	4.28	< .001
100-199% FPL	0.11	1.11	0.00	73.40	< .001	0.121	1.13	0.07	1.70	.090	0.13	1.14	0.09	1.36	.173
200-399% FPL	0.00	1.00	0.00	− 2.02	0.044	0.014	1.01	0.06	0.22	.829	0.02	1.02	0.08	0.22	.824
Highest education in the household: reference level = college and above															
Less than high school	− 0.21	0.81	0.00	− 104.85	< .001	0.016	1.02	0.10	0.16	.872	0.01	1.01	0.14	0.08	.933
High school or GED	0.33	1.39	0.00	245.93	< .001	0.369	1.45	0.06	5.74	<.001	0.35	1.42	0.09	4.10	< .001
Some college or technical school	0.20	1.22	0.00	159.98	< .001	0.348	1.42	0.06	5.87	<.001	0.34	1.41	0.08	4.39	< .001
Child sex: Reference group = Male	− 0.81	0.45	0.00	− 817.93	< .001	− 0.78	0.46	0.05	− 16.63	< .001	− 0.78	0.46	0.06	− 12.46	< .001
Child age	0.10	1.11	0.00	921.20	< .001	0.108	1.11	0.01	19.75	< .001	0.11	1.11	0.01	14.83	< .001

	Between states					Between states					Between states				
Race: reference group = White, non-Hispanic															
Hispanic, non-Hispanic	− 0.83	0.43	0.49	− 1.70	.089	− 0.88	0.42	0.52	− 1.70	.088	− 0.90	0.41	0.60	− 1.49	.136
Black, non-Hispanic	− 0.13	0.87	0.66	− 0.20	.839	− 0.18	0.84	0.66	− 0.27	.785	− 0.20	0.82	0.76	− 0.27	.789
Asian, non-Hispanic	− 1.43	0.24	0.80	− 1.79	.073	− 1.24	0.29	0.87	− 1.43	.153	− 1.22	0.29	1.03	− 1.18	.236
Other/Multi-racial, non-Hispanic	0.13	1.14	0.75	0.18	.858	− 0.08	0.92	0.76	− 0.11	.916	− 0.09	0.91	0.88	− 0.10	.918
Income: reference = 400% FPL or more															
0-99% FPL	2.87	17.60	1.58	1.82	.069	2.388	10.89	1.63	1.47	.143	2.55	12.86	1.86	1.37	.170
100-199% FPL	− 2.17	0.11	1.14	− 1.90	.058	− 2.32	0.10	1.14	− 2.03	.043	− 2.45	0.09	1.30	− 1.88	.060
200-399% FPL	− 0.63	0.53	0.87	− 0.72	.470	− 0.68	0.51	0.92	− 0.74	.461	− 0.66	0.52	1.05	− 0.62	.533
Highest education in the household: Reference level = college and above															
Less than high school	5.48	239.65	3.07	1.79	.074	5.975	393.45	3.26	1.83	.067	6.18	485.29	3.82	1.62	.105
High school or GED	0.80	2.22	1.30	0.62	.538	1.234	3.43	1.27	0.97	.331	1.16	3.19	1.43	0.81	.418
Some college or technical school	0.57	1.77	1.21	0.47	.638	0.635	1.89	1.28	0.50	.621	0.62	1.86	1.50	0.41	.679
Child Sex: Reference group = Male	− 2.73	0.07	1.44	− 1.89	.058	− 1.99	0.14	1.48	− 1.35	.177	− 1.82	0.16	1.72	− 1.05	.292
Child age	− 0.05	0.95	0.13	− 0.40	.687	− 0.02	0.98	0.13	− 0.19	.853	− 0.02	0.98	0.16	− 0.15	.883
RSV	0.02	1.02	0.00	3.63	< .001	0.015	1.01	0.00	3.28	.001	0.01	1.01	0.01	2.80	.005

Model 2O: an unscaled weighted model using the “FWC” variable from the dataset. Model 2A: weights were adjusted by a factor that represents the proportion of cluster size divided by the sum of sampling weights within each cluster. Model 2B: weights were adjusted by a factor that represents the sum of sample weights within each cluster divided by the sum of squared sample weights within each cluster (see Carle (2009), Appendix B)

*FPL* federal poverty level, *GED* General Educational Development, *RSV* Google Trends state-level relative search volume. *Coef* coefficient, *OR* odds ratio. *SE* standard error

## Footnotes

1 In line with recommendations from the methodological literature, for our binary logistic outcome, we set the within-level residual variance equal to ${\pi }^{2}/3$, the variance of a standard logistic distribution when calculating the ICC.
